# Decision-making during trial of labour after caesarean; a qualitative study with gynaecologists

**DOI:** 10.1371/journal.pone.0199887

**Published:** 2018-07-18

**Authors:** Anna L. Rietveld, Christianne J. M. de Groot, Pim W. Teunissen

**Affiliations:** 1 Department of Obstetrics and Gynaecology, Amsterdam UMC, location VUmc, Amsterdam, The Netherlands; 2 School of Health Professions Education (SHE), Faculty of Health Medicine and Life Sciences, Maastricht University, Maastricht, The Netherlands; University of North Carolina at Chapel Hill, UNITED STATES

## Abstract

**Objective:**

The attempt of a woman to deliver vaginally after having had a caesarean in a previous pregnancy is increasingly common in current obstetric practice. During a trial of labour after caesarean, gynaecologists consider whether continuing vaginal birth is safe or, alternately, whether a repeat caesarean is advised. There is large variation in the success rates of women with comparable medical risk factors, requiring better insight in how this assessment is made. As a window of opportunity to intervene in this unexplained variation in practice in specific, and in the globally rising caesarean rate in general, our aim was to increase understanding of gynaecologists’ decision-making during trial of labour.

**Study design:**

We conducted a constructivist grounded theory study, interviewing Dutch gynaecologists. Data collection and analysis were performed concurrently. Initial convenience sampling shifted to theoretical sampling as the study progressed. Data collection continued until theoretical sufficiency was reached. We applied open and axial codes to transcripts of the interviews, and then assembled the axial codes into themes that built up to an emerging theoretical framework.

**Results:**

Nine gynaecologists were interviewed. Data indicated they continuously weighed the chance of a successful outcome of trial of labour against the likelihood of adversities. Patients’ opinions, aspects of progress of labour and gynaecologists’ personal stances regarding trial of labour played a role in the decision-making process; these factors are influenced by organisational affordances and culture. Variation in the assessment of individuals’ chances of success and variable thresholds for a repeat caesarean added to the complexity of the decision-making.

**Conclusion:**

This study pieced together patient-, delivery-, physician- and society-related factors that result in vitally important decisions during trial of labour after caesarean; it reveals the complexity as well as the repetitive patterns involved in this process. Exposing these factors offers opportunities to incorporate the decision-making process in targeted educational interventions, with the aim of modifying the underlying assumptions and concepts in order to reduce practice variation.

## Introduction

The frequency of births by caesarean delivery is rising worldwide. At no point in history have caesarean rates been as high as they are today, varying from 20.9% of all births in less developed regions to 27.2% in more developed regions.[1] Although cesareans are now safer than ever, the surgery is not without risks for mothers nor for their new-borns. Caesarean delivery can be a life-saving procedure, but when compared to vaginal deliveries, mothers delivering by caesarean are more often confronted with infection and haemorrhagic complications, and babies born by caesarean are at higher risk of breathing problems.[2] There is no evidence that a caesarean is beneficial for either a mother or a child if the procedure is not necessary.[3] These data raise the question of how gynaecologists determine, especially during delivery, when a caesarean is needed for the benefit of the mother or the child.

One of the biggest contributors to the rising caesarean rate is the repeat caesarean after a previous one.[4] Pregnant women who have previously had a caesarean are counselled antepartum by their gynaecologist in order to make a decision on the planned mode of delivery. Internationally, guidelines advise incorporating the preferences of the pregnant woman in counselling on the mode of delivery, leaving both options open for discussion so that she has the opportunity to choose the preferred mode of delivery.[5,6] Throughout the Western world, the rates of women opting for trial of labour after one caesarean vary widely, from 20 per cent in the United States to approximately 50 per cent in the United Kingdom and over 70 per cent in the Netherlands.[7–9] In absolute numbers, annually more than 70,000 pregnant women in the US and more than 3,800 pregnant women in the Netherlands opt for a vaginal delivery after a previous caesarean.[7,9]

Due to the globally rising caesarean rate and, consequently, the rise in the number of women who pursue vaginal birth after a caesarean, gynaecologists are increasingly confronted with women in trial of labour after caesarean.[10] Not only does the intended mode of delivery differ geographically, the success rates of trial of labour also differ widely between hospitals. In the Netherlands in 2010, rates of successful trial of labour after caesarean varied between hospitals from 50 to 90 per cent for all women who started a trial of labour, while their identifiable risk factors did not differ.[11] A comparable variation has been described for the US.[12] This suggests that circumstances other than strictly medical risk factors influence the chance of a successful trial of labour. Previous qualitative research on factors of importance for improving the rate of vaginal birth after caesarean has demonstrated the influence of the maternity care system in a country, and the way in which care is offered during pregnancy and birth.[13] However, the fact that success rates differ widely even within countries warrants better insight into how women are managed clinically during trial of labour after caesarean.

In the Netherlands, as in many countries, a resident or clinical midwife looks after a woman during trial of labour after caesarean, supervised by a gynaecologist. The gynaecologist has the final responsibility for advising the labouring woman (and her partner) on whether the vaginal birth attempt can safely proceed or whether a repeat caesarean is indicated: this situation is exemplary of decision-making under uncertain circumstances. Trial of labour after caesarean differs from labour that is not preceded by a caesarean for several reasons, most notably the risk of rupture of the uterine scar. The incidence of uterine rupture during trial of labour after one previous caesarean is estimated to be 0.5 to 1.0 per cent.[7,14] Of all cases of women with uterine rupture, 25 per cent result in severe neonatal and/or maternal morbidity with markedly increased mortality rates.[15] Timely recognition of uterine rupture and a rapidly performed caesarean can reduce these risks. Yet, clinical guidelines include relatively brief paragraphs on intrapartum management[6,16]. They advise the attendance of personnel familiar with the potential complications of delivery after caesarean. However, symptoms of uterine rupture or imminent rupture, such as vaginal blood loss, maternal hypotension or an abnormal foetal heart rate pattern, are unspecific and could have other causes that do not necessarily demand an emergency caesarean.[6] For example, vaginal blood loss might also be normal in the process of delivery, and maternal hypotension can be caused by epidural analgesia. Besides the suspicion of uterine rupture, other reasons to perform a repeat caesarean during trial of labour can be lack of progress and suspected or imminent foetal distress. Determining whether or not a labour should be considered prolonged proves to be arbitrary and analysis of the foetal heart rate pattern is subject to intra- and inter-observer disagreement; these factors add to the uncertainty in deciding whether or not to perform a caesarean during trial of labour after a previous caesarean.[17,18]

The way in which obstetric healthcare professionals in general manage their patients has been the subject of international study, revealing that fear of medico-legal consequences is a major factor prompting risk-avoidance behaviour.[19–22] Research on the specific subject of the clinical management of trial of labour after caesarean is scarce.[5] In 2015, Yee et al. focused on personality traits of American gynaecologists, showing that low physician anxiety increased the chance of vaginal birth without increasing the chance of uterine rupture.[23] However, previous research on clinical decision-making in general has described that the medical decision-making process is affected not only by intrinsic personality traits of caregivers, but also by extrinsic factors such as the characteristics of the patient and the clinician’s interaction with his profession and the health care system.[24,25]

In order to understand why women with comparable risk factors have different chances of vaginal delivery after caesarean, we need to go one step back to explore the decision-making process during trial of labour after caesarean, taking a broader perspective than focusing only on specific personality traits. This study aims to explore how gynaecologists responsible for women during their trial of labour after caesarean reach decisions regarding whether to advise continuing labour or to perform a caesarean. Knowing more about the decision-making process during trial of labour after caesarean could make medical staff aware of influential factors. These factors could then be incorporated in training programmes for current and future gynaecologists in order to improve the decision-making that leads to advising women before and during trial of labour after caesarean. The aim here is to reduce practice variation and to open opportunities to intervene in the globally rising caesarean rate.[26]

## Methods

We used a constructivist grounded theory approach. Constructivist grounded theory is a qualitative research method appropriate if the phenomenon under study has not yet been explained by an existing theory. The constructivist grounded theory technique is an iterative process of data collection and analysis.[27] We chose to collect data by individual, in-depth, semi-structured interviews to gain a rich insight into the decision-making process. Recruitment, data collection and analysis took place between April 2016 and March 2017.

### Setting

We recruited gynaecologists from the Netherlands, where maternity care is organised in a primary, secondary and tertiary care model. Primary care, for low-risk women, is performed by primary care midwives and general practitioners. Secondary, clinical care consists of gynaecologists, residents and clinical midwives in general hospitals, and tertiary clinical care comprises gynaecologists, residents and clinical midwives in academic hospitals.[28] All residents and clinical midwives work under the supervision of a gynaecologist. Because of the risk of uterine rupture, women with a previous caesarean deliver in clinical, secondary or tertiary, care. They are consequently attended by residents or clinical midwives under the supervision of a gynaecologist. This means that gynaecologists are ultimately responsible for decisions concerning trial of labour after caesarean. The Dutch setting was suitable for answering our research question because trial of labour after caesarean is relatively common in the country. Women are not looked after by only one gynaecologist during pregnancy and delivery, but are cared for by a team of midwives, residents and gynaecologists working as a unit in a hospital. This means that a woman’s antenatal visits may have been with different gynaecologists and the gynaecologist on call might meet a woman for the first time during her delivery. It is therefore quite likely that a woman’s motivation to opt for trial of labour may not have been discussed with the gynaecologist attending her delivery.

### Participants and recruitment

We recruited Dutch gynaecologists of different ages, gender, years of working experience, residency programmes, working environments and subspecialties. Recruitment was done by email and telephone invitation, and participation was voluntary. Participants read the study information leaflet and signed for informed consent before starting the interview. They were made aware of the fact they could discontinue the interview or withdraw from the study at any time. We started with convenience sampling, recruiting gynaecologists in the professional network of the research team. Thereafter, we proceeded to theoretical sampling by inviting gynaecologists with features different from those of the gynaecologists that had already been interviewed, aiming to obtain a variety of viewpoints.[27] Participant recruitment ended when theoretical sufficiency was reached, meaning no changes to the emerging theory had to be made based on the interviews.

### Data collection and analysis

As presented in the S1 Interview guide, the semi-structured interviews were conducted using an interview guide which served as a tool to support interviewing, rather than a strict checklist. The open-ended questions were intended to encourage gynaecologists to describe their behaviour, reasons and rationales in caring for a patient in trial of labour after caesarean. The interviews were conducted at a place convenient to the participant (five times at the hospital where the participant worked, twice at the participant’s home, once at the investigator’s workplace, and once using Skype). The interviews were conducted privately. Interviews were audio recorded, transcribed verbatim and anonymised. We employed a web-based qualitative data analysis system, Dedoose (version 7.5.14, Los Angeles, CA: SocioCultural Research Consultants, LLC), to code and extract data. In line with constructivist grounded theory methodology, data collection and analysis were performed simultaneously, allowing new issues or themes to emerge in the subsequent interviews. A.R. and P.T. coded the first two interviews together. In instances where a different code was applied to the same citation, consensus was reached by discussion. Questions that arose when coding the interviews and that remained unanswered were introduced in subsequent interviews. Data were first converted into initial codes by means of line-by-line coding. Thereafter, focused codes were developed from the initial codes. These focused codes were then merged into conceptual themes. Following further analytic discussion with A.R., C.G. and P.T., a model was constructed that accounted for the relationship among the themes identified.

### Research team and reflexivity

One of the investigators (A.R.) conducted the interviews. At the time of the interviews, she worked as a house officer in obstetrics and gynaecology and was a PhD student at Amsterdam UMC, location VUmc. Apart from the first participant, who was a former colleague, she did not know or work together with any of the participants. We considered the interviewer’s field experience as an advantage facilitating in-depth interviewing in this topic. In order to avoid the pitfall of the interviewer assuming she knew what participants meant based on common knowledge of clinical practice, participants were asked not to expect the interviewer to understand everything they told her. The interviewer regularly asked for an explanation or reasoning, even when she thought she knew why a participant performed a certain action or had a particular opinion. This strategy encouraged participants to explain their thoughts and opinions. P.T. is a perinatologist at Amsterdam UMC, location Vumc and a professor in medical education at Maastricht University, his focus being workplace-based learning. C.G. is a perinatologist and a professor in obstetrics and gynaecology at Amsterdam UMC, location VUmc. Her main research focus is high-risk pregnancy, especially hypertensive disorders in pregnancy. We recognised the potential for the team to introduce their own perspectives into the analysis, based on their own experiences in clinical practice. Reflexivity was practised by checking the emerging theoretical framework with the initial codes. When a discrepancy was noted, meaning an initial code did not match one of the themes in the framework, the discussion was referred back to the team to discuss whether changing the emerging framework was warranted.

### Ethical approval

The Medical Ethics Review Committee of Amsterdam UMC, location VUmc examined the study protocol (#2016.143) and judged that an official approval of this study was not required. Informed consent in writing was obtained from every participant.

## Results

**Fig 1:**
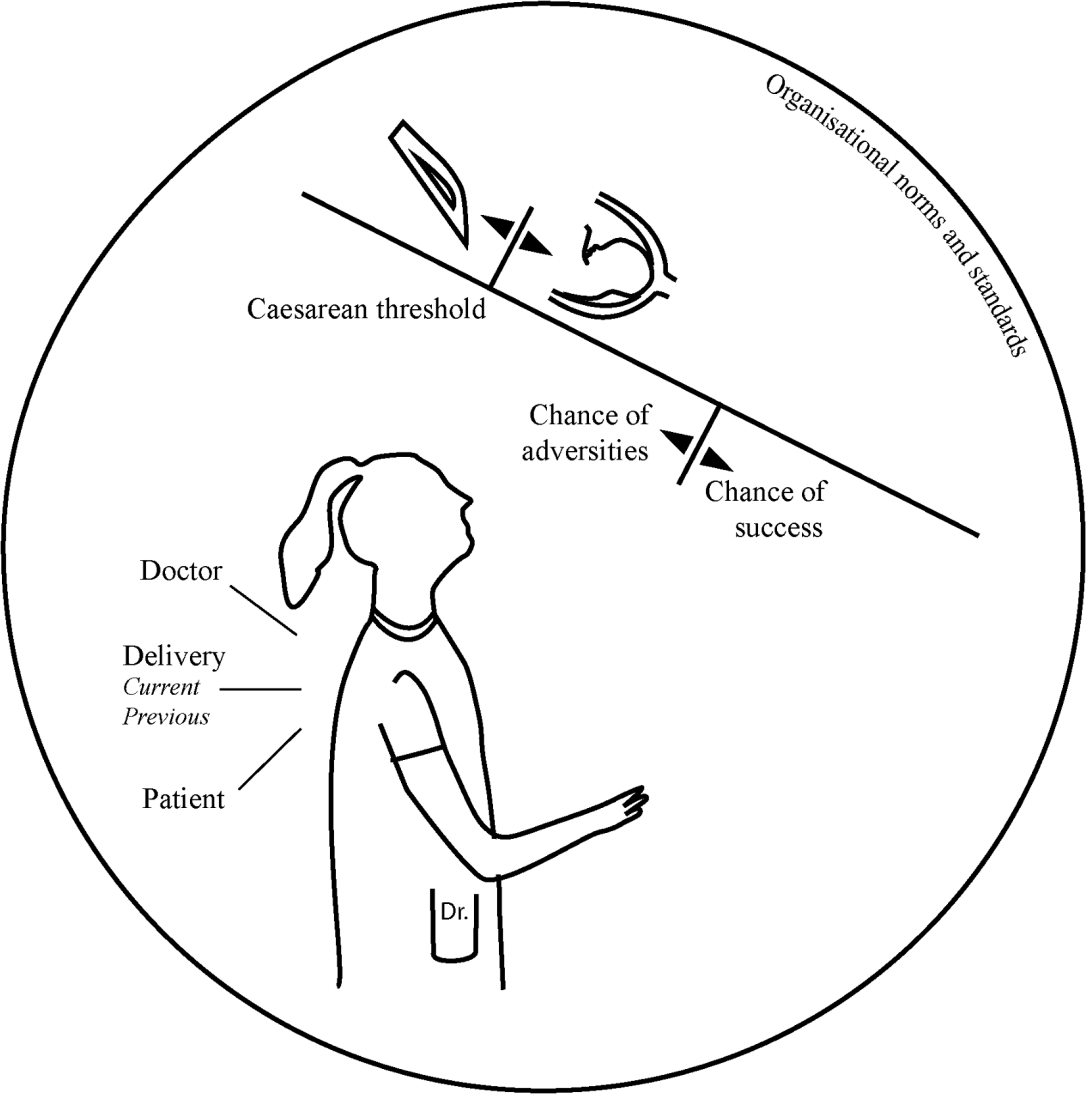
Model of gynaecologists’ decision-making during trial of labour after caesarean.

Nine gynaecologists working in nine different hospitals in six provinces in the Netherlands were included in the study. Two gynaecologists from two other hospitals declined participation due to lack of time. Table 1 shows the characteristics of the participants. In the interviews concerning trial of labour after caesarean, participants referred to situations they had experienced or to fictive situations they might encounter at some point in time. They explained what thoughts they had about the way they acted and their considerations in other, sometimes hypothetical, scenarios. The participants acknowledged the fact that, although they had final responsibility for a woman’s trial of labour, they were dependent on the team of nurses, clinical midwives and residents they were working with for most of their clinical information. They reflected on themselves as members of the care team, and the possibility of their being influenced by the individuals with whom they worked within the team. A theoretical framework (Fig 1) was constructed during the iterative process of constructivist grounded theory. After seven interviews the framework appeared complete, covering all aspects that arose from the interviews. To test the applicability and completeness of the framework, two more interviews were conducted. No new data arose from these interviews, indicating data saturation. The main underlying assessment participants dealt with appeared to be: when is continuing with a trial of labour after caesarean still acceptable and when is it not, meaning one proceeds to a caesarean?

**Table 1:** Participants’ characteristics.

Age	Gender	Hospital type	Years^a^	Subspecialty
36	F	Teaching non-academic hospital	2.5	Obstetrics
37	F	Teaching non-academic hospital	0.5	Gynaecology
38	F	Teaching academic hospital	3.5	Obstetrics
38	F	Non-teaching non-academic hospital	3.5	Gynaecology
40	M	Teaching non-academic hospital	4	Gynaecology
42	M	Teaching academic hospital	8	Gynaecology
49	F	Teaching academic hospital	16	Obstetrics
54	M	Non-teaching non-academic hospital	18	Gynaecology
58	M	Teaching academic hospital	24	Obstetrics

^a^ Years as a consultant

In this results section, we refer to the point at which the decision is taken to perform a repeat caesarean during trial of labour as the caesarean threshold. Once the threshold has been passed, gynaecologists indicated that they switch to a different mode. This mode entails arranging the caesarean, which requires communication and collaboration with patient, partner and other health professionals, such as the anaesthesiologist. They generally do not return to the mode of (re-)assessing whether a vaginal delivery might still be an option. Two mechanisms play a role and their interaction determines whether or not the caesarean threshold is crossed. The first is a process that consists of the interplay between doctor, patient and the delivery itself in a comparative assessment of the likelihood of a successful vaginal birth versus the likelihood of adversities, bearing in mind the organisational norms and standards. The second mechanism is that of the situational variability of the caesarean threshold. The threshold is not a fixed benchmark, but appears to be influenced, among other things, by what is accepted within the organisation where one is working.

We will explain these two interacting mechanisms influencing clinical decision-making in trial of labour after caesarean in the following section, referring to them as the ‘comparative assessment’ and the ‘variable caesarean threshold’. Fig 1 represents the core concepts and their interactions. In the following text, quotes illustrate the complex decision-making process during trial of labour after caesarean. Quotes are tagged with letters referring to the participant to whom the quotes belong. However, in order to maintain participant confidentiality, they do not correspond to the order in which participants are presented in Table 1.

### Comparative assessment

#### Chance of success versus chance of adversities

Gynaecologists explained that, in assessing whether trial of labour after caesarean was still acceptable, they were required to make a continuous comparative assessment between the perceived chance of success and the perceived chance of an adverse outcome. The participants explained that how they perceive a particular situation is often influenced by the chance they think a patient has of delivering vaginally.

*“Well…*. *If you have a foetal heart rate pattern of which you think*: *‘Well… well…*., *not great’*, *and she had only a 20% chance of delivering vaginally to begin with*, *then*, *yes*, *you will assess it differently from if she had an 80% chance of delivering vaginally*.*”* (Dr H.)

Participants assessed the chance of success at different points in time. Some do this at the start of labour, and some do so only when a problem occurs.

*“In this way*, *we estimate how much attention we need to pay to this person and what I think the chance of her delivering vaginally is*. *And I keep that in mind*.*”* (Dr C.)

*“I think [the estimation is made]*, *when there’s something wrong*, *when a plan has to be made*, *when things don’t follow the normal course of labour*. *That is*, *when the foetal heart rate is non-reassuring or when the patient is in pain*, *that kind of stuff*.*”* (Dr B.)

The estimation of the chance of success is characterised as a double-edged sword. It comprises an assessment of the chance of success versus the potential risks, while at the same time this initial assessment influences the way in which subsequent events are viewed. It can, for instance, prompt defensive strategies when the chance of success is perceived to be fairly low.

*“[It can be] dangerous*, *it could become a self-fulfilling prophecy; at the point that you are a bit less positive*, *then you might perform that caesarean earlier*, *meaning that the patient is not getting a chance [to deliver vaginally]*.*”* (Dr F.)

### Influences on the comparative assessment

The way in which the comparative assessment is made is not a straightforward calculation of positive and negative contributors to delivering vaginally, resulting in an unchangeable percentage. Rather, our analysis showed it is an evolving process, influenced by three interacting, situationally dependent factors: the doctor, the patient and the delivery itself. The participants constantly evaluated the progress of delivery in relation to what they thought and how the patient felt about it.

#### The doctor as influential factor

The participants saw it as their responsibility to prevent ending up in a situation where they had to perform a repeat caesarean in an acute setting, either for maternal or foetal reasons. They acknowledged that there is a certain ‘number needed to treat’ to prevent one adverse maternal or neonatal outcome, meaning that they accepted performing several repeat caesareans at a relatively early stage in order to prevent performing one late, or even too late. During trial of labour after caesarean, they indicated that they did not want to push the limits.

*“The bar to perform a caesarean is set really low*. *[…] Normally you would step on the gas if you see an orange traffic light*, *but if the patient had a caesarean before*, *you hit the brakes*.*”* (Dr B.)

What those limits are, however, varies per gynaecologist. Experience, as well as age, seemed to play a role. The way in which gynaecologists are trained seemed to be of lesser importance, being overruled by the changing zeitgeist.

*“I think so many things have changed in the last ten to fifteen years that these changes have more influence than the place you did your training.”* (Dr G.)

Another, rather intangible factor that influenced the comparative assessment was described as ‘the doctor’s gut feeling’. The participants stated that they learned to follow their intuition.

*“It [the advice given] will always be to some extent the personal taste of the gynaecologist*, *and that’s not necessarily a bad thing*.*”* (Dr E.)

#### The patient as influential factor

Patient-related factors that played an important role in the continual comparative assessment relate to the patient’s motivation for vaginal birth.

*“But in the whole ‘vaginal birth after caesarean thing’*, *it [the patient’s wish] plays a role because*, *in fact*, *the patient can choose*. *So*, *I take the wishes of the patient more and more into account*, *compared to twenty years ago*.*”* (Dr A.)

In counselling during labour, doctors described listening to the patient’s wishes, rather than prescribing what was deemed to be best for the patient.

*“Because I think the patient’s motivation is very important*. *The patient needs to be motivated and she must clearly not have the feeling that she’s forced into a vaginal delivery*. *If things do go wrong*, *we have to be able to look one another in the eye and say quite firmly*: *We had a plan together*, *a well-defined plan*.*”* (Dr C.)

Not only the antepartum motivation, but also the intrapartum opinion or change of opinion of the patient played an important role in participants’ decision-making process.

*“If she says*: *‘I see it differently now and I actually want a caesarean*, *because (…) I don’t want to take the risk or it doesn’t feel good’*, *then*, *I would discuss the risks and benefits one more time and if she wants a caesarean*, *that’s what I will do*.*”* (Dr H.)

Participants referred to the psychological wellbeing of their patients, and paid a great deal of attention to a patient’s state of mind. They were concerned with the experience their patients have during the delivery and how they will look back on it.

*“Of course*, *you want people to have a great birthing experience*. *So*, *in that sense you want to try to make this second delivery a bit more successful [than the first one]*. *And so…*. *Yes*, *I think… I think I would more readily decide to go for a caesarean*, *and if it leads to this great birthing experience*, *then I believe it is a wise decision*.*”* (Dr B.)

#### The delivery as influential factor

The reasons mentioned by participants for performing a repeat caesarean during trial of labour are non-progression in either the first or the second stage, or suspected foetal distress. A mechanism that played a role in the comparative assessment of the chance of success versus the chance of adversities is to relate the course of the current labour to the problem that arose in the previous birth, leading to the first caesarean. If a woman has previously experienced labour dystocia, the balance in the comparative assessment is likely to tip at an earlier stage towards a lower perceived ‘chance of success’ as compared to women who underwent a planned caesarean because of a breech position. The adversity which participants tried to forestall is uterine rupture. They expected, based on their knowledge of the literature, that the chance of uterine rupture would increase if they were to medically augment contractions when there is persistently not enough progression in labour.

*“It also depends on the last delivery; if a woman who didn’t get past 6 centimetres of dilation last time seems to come to a halt at 6 centimetres again*, *I will say we really need more dilation soon or it seems that history will repeat itself and then that leaves us with no choice*.*”* (Dr G.)

Although participants describe non-progression as a stronger incentive to perform a repeat caesarean, the suspicion of foetal distress also leads to the caesarean threshold being reached.

*“Of course*, *we know that changes in the foetal heart rate pattern might be the only first sign of uterine rupture*. *So*, *at the point when you consider [getting more information on the foetal condition by performing] foetal blood sampling on a woman who has already had a caesarean*, *you have to ask yourself if that is the right way to go*, *or if it means you simply have to perform a repeat caesarean*, *because you don’t trust the condition of the baby and that is potentially the first sign of uterine rupture*.*”* (Dr D.)

One factor straightforwardly leading to the caesarean threshold being reached, independently of patient- or doctor-related factors, was persistent foetal bradycardia. Bradycardia was often described as a situation leaving little room for contemplation or clinical evaluation, although not always immediately raising the suspicion of uterine rupture.

*“She had persistent bradycardia*. *[…] There was no choice*. *[…] There was no suspicion of uterine rupture*, *there just was a foetal problem*.*”* (Dr A.)

One of the participants added the importance of ruling out certain mechanisms that can cause foetal bradycardia, such as uterine hyperstimulation or maternal hypotension, opening up opportunities to continue with vaginal delivery. Nevertheless, she did this with a great deal of caution, by initiating a parallel action to already prepare for an emergency caesarean.

*“But if she had not previously had a caesarean*, *I would not have signed her up for theatre yet*.*”* (Dr I.)

### The variable caesarean threshold

Based on the participants’ explanations, a concept we refer to as ‘the variable caesarean threshold’ emerged. This describes the threshold an individual gynaecologist applies for deciding on the acceptability of continuing with trial of labour. If the comparative assessment of the chance of success versus the chance of adversities crosses this line, it leads to the decision to advise proceeding to a repeat caesarean. There appeared to be multiple reasons why gynaecologists crossed the caesarean threshold, such as suspicion of foetal compromise, lack of progression of labour or the patient’s wishes, as explained above. Although these reasons may seem to lead to clear cut-offs for opting for a repeat caesarean, these decisions are not equivocal. The variability of the threshold played an important role here and appeared to stem from sociocultural aspects, as explained by the differences per hospital and by differences over time.

#### Variability of the caesarean threshold caused by place of work

The caesarean threshold seemed to be dependent on the medical staff, the local facilities and the organisational culture.

*“I did a lot of caesareans in an academic hospital*, *about which I think now*, *when looking back*, *that if the same patients were to deliver with me in my current non-academic hospital*, *I would perform significantly fewer caesareans*.*”* (Dr C.)

The physical working environment influenced the threshold to perform a caesarean. The availability of a surgical team (including an anaesthetist) is something gynaecologists bore in mind during trial of labour.

*“So*, *you have to keep in mind*, *very clearly*: *the moment I decide ‘This is not good*, *I want to do a caesarean’*, *it takes time to actually perform the caesarean*. *In other words*, *you have to decide sooner rather than later*.*”* (Dr C.)

Gynaecologists described the importance of uniformity in policy at their place of work

*“For example*, *if no one will augment labour during trial of labour anymore*, *then I’m also not going to do that*. *Or if women who’ve had a previous caesarean are not allowed to have pain relief anymore*, *then who am I to still give it*?*”* (Dr B.)

The participants acknowledged that this sometimes needed personal adjustment. For example, Dr H., who had just switched hospitals, encountered a different approach towards trial of labour in her new working environment, experiencing that the caesarean threshold was relatively high compared to her former workplace.

*“You have to work together*. *So*, *I have to compromise*.*”* (Dr H.)

The caesarean threshold was influenced not only by written protocols, but also by a collective sense of fear of medico-legal consequences.

*“It actually is the reason we are so cautious*, *not because we have had a disciplinary case ourselves*, *but because we have colleagues who have been involved in the disciplinary board as expert witnesses*, *who see these cases with bad outcomes*, *so it has definitely influenced our policy*.*”* (Dr F.)

#### Variability of the caesarean threshold caused by societal changes

Besides the local situation, participants stated that society influenced the caesarean threshold.

*“Yes [the threshold to perform caesarean during trial of labour after caesarean is lower nowadays]*, *because numerous indications to perform caesareans have emerged; the delivery after a caesarean*, *the breech position*, *where it is legitimate to perform caesarean for these reasons*, *meaning that you also include the grey area surrounding it*. *So*, *you see the number of caesareans rising (…) because we perform caesareans for less strict indications*. *In the past*, *the indications were very strict*, *and now they’re…*, *well they’re starting to become somewhat*, *weaker*. *(…) Similarly*, *the ‘birth after caesarean’ guideline is not purely based on scientific evidence*, *but above all on societal changes*.*”* (Dr A.)

Participants described a shift in what is deemed to be a ‘normal’ delivery, noticing that a caesarean has increasingly become a well-accepted means of childbirth, as compared to, for example, twenty years ago.

*“That is such a long time ago*, *it was a different time back then*. *I can remember it; everybody delivered vaginally*.*”* (Dr F.)

Participants attributed this change to the perceived safety of caesarean delivery and the relatively quick recovery, as well as the fact that patients are becoming increasingly better educated, not seeing the doctor not as an authority but more or less as a negotiating partner, including during delivery.

*“I try to involve women*, *as far as possible during delivery*, *in the pros and cons of one or the other choice*, *more than a number of years ago*. *(…) I think people have become more outspoken; they no longer assume that the doctor knows best*. *(…) Now we have a discussion and you inform the patient extensively*, *but if she still wants a caesarean*, *she will have one*.*”* (Dr G.)

## Discussion

Our study was designed to explore how gynaecologists make decisions during trial of labour after caesarean. Internationally, guidelines consider trial of labour after caesarean as a reasonable way to bring the globally rising caesarean rate to a halt, despite this carrying the risk of a repeat caesarean due to foetal distress, uterine rupture or non-progression, or suspicion of any of these.[6,10,16] In view of the practice variation among gynaecologists and among hospitals, greater insight is required into the process of decision-making during trial of labour after caesarean.[11]

Our results showed that during a trial of labour gynaecologists continually assess and re-assess the chance of success versus the chance of adversities. This assessment is related to the patient, the current and previous delivery and their own stance, while at the same time being subject to a socio-culturally influenced threshold that tacitly defines when to perform a caesarean.

The idea that not only biomedical influences but also sociocultural factors play a significant role in medical decision-making is not a novelty; it has, however, not been extensively explored in the specific case of intrapartum decision-making during trial of labour after caesarean.[25] Kamal and colleagues explored antepartum factors influencing repeat caesarean rates, addressing the fact that decision-making is a social practice that does not necessarily benefit from standardised protocols and medico-technical knowledge.[29] Yet, research aimed at reducing the risks associated with multiple caesareans has focused on implementing clinical guidelines to promote the success of vaginal birth.[30] Our study underlines the fact that it will not be enough to focus on providing clear clinical guidelines. Guidelines might influence the caesarean threshold, but are unlikely to influence the process of reaching this threshold. Our results indicate that gynaecologists and gynaecology residents should be made aware of the mechanisms that play a role in decision-making during trial of labour after caesarean, such as those we found in the current study. Incorporating our framework into targeted educational interventions may well enhance gynaecologists’ ability to reflect explicitly on crucial factors in decision-making. For example, if a caesarean is performed due to suspicion of uterine rupture, the structured and explicit evaluation of the fact that a rupture was indeed present (or not) can be used to optimise the comparative assessment. This may result in underlying assumptions and concepts being adjusted and unexplained variations in practice being reduced.[31] Our results point to a window of opportunity for educational interventions not only during residency training, but also for registered gynaecologists who have already completed their training.

Our framework can be used in antepartum counselling of women who are pregnant after a caesarean in order to explain how a trial of labour is conducted. In current practice, antepartum counselling focuses on discussing the possible risks and benefits of both trial of labour and planned repeat caesarean, often complemented with the patient’s personal wishes and considerations on family planning.[6] Practical guidelines do not advise gynaecologists to include information on the possible course of labour in antepartum counselling, let alone mentioning the subjectivity of a caregiver or caregivers attending the delivery. Our findings provide insight into this subjective process, opening up a discussion on how this should be reflected in antepartum counselling. Moreover, our framework shows the importance of further research on patients’ decision-making and on the values that underlie organisational standards, since these aspects act upon gynaecologists as decision-makers.

There are limitations in drawing conclusions based on gynaecologists’ self-reported insights in their decision-making. A gynaecologist might, for example, give socially desirable answers. None of the participants brought up the fact that decisions might be influenced by time of day (or night).[32] However, this is difficult to test since in-vivo observation of gynaecologists’ behaviour, including on-the-spot questioning why they are doing what they are doing, will seriously disturb the clinical workflow. The purpose of this study, however, was to understand gynaecologists’ perceptions of the decision-making process. Future studies could add other stakeholders’ perspectives. A second limitation of this study might be a selection bias. Gynaecologists who regard vaginal birth after caesarean more favourably might have been more likely to agree to participate in this study. Nonetheless, only two gynaecologists declined participation. Theoretical sampling was used to eliminate a selection bias as much as possible.

Constructivist grounded theory does not claim to produce generalisable truths.[27] We constructed a rather broad theoretical framework that we think is transferable to other contexts, drawing on maximizing variation in age, gender and subspecialty. However, all our participants were Dutch gynaecologists. The exposure to trial of labour after caesarean for Dutch gynaecologists is relatively high compared to their peers in several other countries. Further research is needed to determine the transferability of our findings to gynaecologists in different countries. However, we do not expect the framework to change drastically by country, since, although the organisational norms and standards and possibly the doctors’ personal beliefs might differ, they will still be likely to influence decision-making.

## Conclusions

Gynaecologists’ decision-making during trial of labour after caesarean is an example of medical decision-making under uncertain circumstances. This qualitative study brought together the patient, delivery and physician as well as organisational and societal factors that result in vitally important decisions, revealing the complexity as well as the repetitive patterns in decision-making during trial of labour after caesarean. Exposing these factors opens up opportunities to incorporate the decision-making process in targeted educational interventions. A structured and explicit evaluation of intrapartum events may adjust underlying assumptions and concepts, contributing to reducing the unexplained practice variation in trial of labour success rates.

## Supporting information

S1 Interview guide Guide for the semi-structured interviews. (DOCX)
