# Supplementary material for: Decision-making during trial of labour after caesarean; a qualitative study with gynaecologists
Source: PLoS One. 2018 Jul 18;13(7):e0199887. doi: 10.1371/journal.pone.0199887 (PMC6051596; doi:10.1371/journal.pone.0199887)
Supplement: S1 Interview guide — (DOCX) [file pone.0199887.s001.docx]

**Supporting information**

**S1 Interview guide. Guide for the semi-structured interviews.** This list was used as guidance for the interviews, but does not present an exclusive topic list. Additional questions were added as the course of the interview requested.

1. Can you describe a recent case of a women in trial of labour after caesarean about whom you were consulted?
2. What is important to you during the clinical management of women in trial of labour after caesarean?
3. Is a trial of labour after caesarean different from labour that is unprecedented by a caesarean? Why (not)?
4. Did you once encounter a situation in which you were in doubt on what to do? Can you tell me about the situation and your considerations?
5. What do you consider reasons to advice to perform repeat caesarean during trial of labour after caesarean? Can you explain why?
